# Using Nearest-Neighbor Distributions to Quantify Machine Learning of Materials’ Microstructures

**DOI:** 10.3390/e27050536

**Published:** 2025-05-17

**Authors:** Jeffrey M. Rickman, Katayun Barmak, Matthew J. Patrick, Godfred Adomako Mensah

**Affiliations:** 1 Department of Physics, Lehigh University, Bethlehem, PA 18015, USA; 2 Department of Materials Science and Engineering, Lehigh University, Bethlehem, PA 18015, USA; goa223@lehigh.edu; 3 Department of Applied Physics and Applied Mathematics, Columbia University, New York, NY 10027, USA; kb2612@columbia.edu (K.B.);

**Keywords:** microstructural segmentation, convolutional network, neighbor distribution

## Abstract

Machine learning strategies for the semantic segmentation of materials’ micrographs, such as U-Net, have been employed in recent years to enable the automated identification of grain-boundary networks in polycrystals. For example, most recently, this architecture has allowed researchers to address the long-standing problem of automated image segmentation of thin-film microstructures in bright-field TEM micrographs. Such approaches are typically based on the minimization of a binary cross-entropy loss function that compares constructed images to a ground truth at the pixel level over many epochs. In this work, we quantify the rate at which the underlying microstructural features embodied in the grain-boundary network, as described stereologically, are also learned in this process. In particular, we assess the rate of microstructural learning in terms of the moments of the *k*-th nearest-neighbor pixel distributions and associated metrics, including a microstructural cross-entropy, that embody the spatial correlations among the pixels through a hierarchy of *n*-point correlation functions. From the moments of these distributions, we obtain so-called learning functions that highlight the rate at which the important topological features of a grain-boundary network appear. It is found that the salient features of network structure emerge after relatively few epochs, suggesting that grain size, network topology, etc., are learned early (as measured in epochs) during the segmentation process.

## 1. Introduction

Many metals and alloys that are employed in engineering applications are polycrystalline, meaning they comprise a multitude of small crystallites that are misoriented with respect to one another and meet at defects called grain boundaries. The grains and grain-boundary structures of materials, known collectively as the microstructure, often dictate the properties and observed performance, including the mechanical (e.g., strength and ductility), electrical (e.g., resistivity), and transport (e.g., diffusion) behavior [1,2,3,4,5,6,7,8]. Therefore, it is extremely important to accurately characterize the microstructural topology. To achieve this aim, one early approach employed edge detection algorithms to segment micrographs into constituent grains and grain boundaries [9]. Unfortunately, due to the complex contrast present in diffraction-contrast images, the segmentations generated by such approaches are missing many true boundaries, where the contrast between grains is not well defined, and are rife with spurious boundaries due to the internal contrast in grains with bend contours, where contrast is present inside a single grain due to the distortion of the crystal planes. More recently, a machine learning approach to this problem has leveraged boundaries in hand-labeled micrographs to train a model based on the U-Net architecture to effect automated grain-boundary segmentation. This strategy has been successfully applied, in particular, to bright-field transmission electron microscopy (TEM) micrographs for the identification of grain-boundary networks [10]. One example of such applications is the recent work of Patrick et al. [11], who employed a supervised learning approach based on a U-Net convolutional neural network to automate the tracing of grain-boundary networks in aluminum, platinum, and palladium thin films.

While a variety of deep learning architectures are available for image segmentation, many microstructural quantities of interest, particularly those related to triple-junction statistics, require precise segmentation of fine features. Furthermore, this task requires models that can learn robust relationships within limited datasets, as the manual labeling of grain-boundary networks is particularly laborious. U-Net is specifically designed to address these challenges in the context of biomedical images. Furthermore, preliminary results suggest that instance segmentations like those generated by YOLOv8 [12,13] and its family of models perform well on grain-size statistics, but the boundary locations and the network’s topology are only known implicitly, and the performance of the models has generally been found to be sensitive to magnification. Together, these findings highlight U-Net as an attractive and relevant architecture for the analysis presented in this paper.

As with other deep learning algorithms, the U-Net algorithm is designed to learn objective (i.e., loss) functions. In this context, the task of image segmentation can be regarded as classification at the pixel level, in which semantic segmentation involves the minimization of a loss function [14]. For most applications, the binary cross-entropy loss function is widely used for segmentation. However, given the centrality of the loss function in the segmentation process, various researchers have proposed alternative functions in different application domains to optimize results. These alternatives can be categorized based on their functional form, notably including those comparing underlying probability distributions (e.g., cross-entropies) and those comparing the distance between the predicted structure and the ground truth (e.g., distance loss metrics) [14,15].

In this work, using U-Net as a test case, we address the question: What is being learned during deep learning-based segmentation of a material’s microstructure? In particular, we seek to highlight the rate at which the underlying microstructure, as reflected in the topology of the grain-boundary network, is learned when a pixel-level objective function, such as the binary cross-entropy, is minimized. This is accomplished by defining so-called learning functions that embody the spatial correlations among the pixels that comprise the grain-boundary network. These functions are based on the moments of the *k*-th nearest-neighbor pixel distributions, as these distributions can be expressed through a hierarchy of *n*-point correlation functions that reveal the spatial arrangement of the pixels in the boundary network. As demonstrated below, it is found that the structure of this network emerges after relatively few epochs in the segmentation process.

## 2. Materials and Methods

To test our methodology, we interrogate microstructures acquired from samples imaged using bright-field transmission electron microscopy (BF TEM). The grain-boundary network obtained from these images is constructed using U-Net and subsequently analyzed using the spatial distribution of the neighbors of the network pixels. The associated experimental and data-analytic methodologies are described below.

### 2.1. Experimental System and Imaging

The microstructures under study were generated from a 100 nm thick aluminum thin film that was sputter deposited on a Si(100) with a 300 nm thick thermal oxide [16]. The substrate was rotated at 20 rpm to ensure deposition uniformity using a DC magnetron sputtering system (with a base pressure in the $10^{-9}$ Torr range) that was operated at 500 W DC using 1.1 mTorr of purified argon (99.9995%). After this procedure, the sample was cut into square chips (<1.6 mm × 1.6 mm), which were subsequently annealed at 185 °C in a reducing atmosphere of flowing Ar/H_2_. It was found that the grain-size distributions were equivalent for the different annealing times considered here. These chips were first mechanically thinned to <200 μm, and then chemical back etching was employed to remove both the silicon wafer material and some of the thermal oxide to yield large (∼1 mm diameter) electron-transparent regions. The resulting samples were imaged via BF TEM in an FEI F200X Talos S/TEM (200 kV accelerating voltage, 30 μm objective aperture, 36,000× magnification). Images were recorded for three sample tilts per field of view on a 4096×4096 BM-CETA charge-coupled device (CCD) camera and were subsequently downscaled to 512×512 during U-Net inferencing. All three sample tilts acquired in this manner were used to inform the manual reconstruction of the microstructures for comparison with the corresponding U-Net reconstructions.

### 2.2. U-Net Implementation

The U-Net architecture was first employed for the segmentation of TEM images of stained biological samples [17,18] and, as noted above, has been used more recently to enable the automated identification of grain-boundary networks in polycrystals [10,16]. Ronneberger et al. [18] developed this “fully convolutional network” architecture and associated training strategy, in which a contracting network is supplemented by layers that enhance the resolution of the output. More specifically, the architecture comprises both compressive (encoder) and expansive (decoder) paths, with the former consisting of two repeated convolutions, a subsequent rectified linear unit (ReLU), and a pooling operation, and the latter consisting of a feature map upsampling, a concatenation with a cropped map, and several convolution/ReLU combinations. Further details, including the use of training sets, can be found in the work by Ronneberger et al. As noted above, the algorithm seeks to minimize a binary cross-entropy loss function based on a soft-max operation [19] on a feature map. The binary cross-entropy loss function, denoted here by $l\left(t\right)$, is employed in binary classification problems, such as those performed with U-Net, to quantify the difference between a class label and a model’s predicted probability of that label. This loss function operates at the pixel level to classify a given pixel, thereby effectively quantifying the distance between the probability distributions of the predicted and actual pixel values. By minimizing $l\left(t\right)$, one seeks to improve model accuracy relative to the ground truth. Our aim here is to compare $l\left(t\right)$ with other learning measures, as defined below, that highlight the rate at which a grain-boundary network is constructed during U-Net processing.

**Figure 1:**
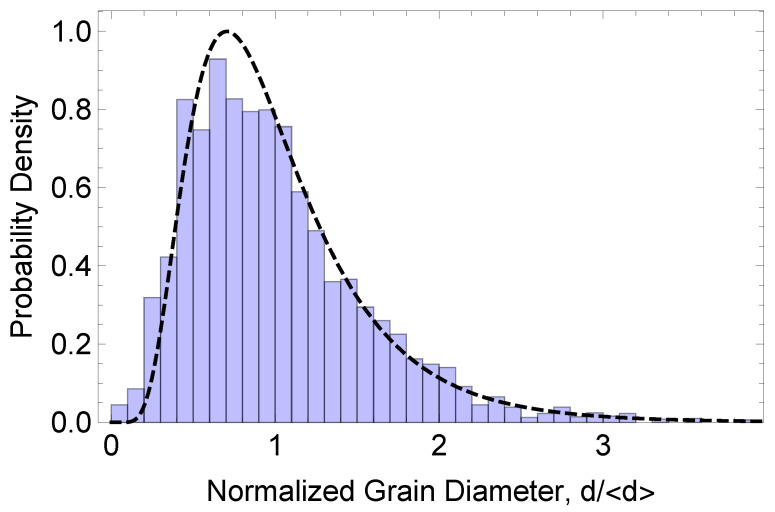
The probability density function of the normalized effective circular-grain diameter, d/〈d〉, for the training set. Also shown is a fit to a log-normal distribution (dashed line).

The training set used for segmentation in this study comprised images from 299 fields of view. The corresponding grain-size distribution, the probability density function of the normalized effective circular-grain diameter d/〈d〉, is shown in Figure 1 along with a fitted log-normal distribution. As the log-normal distribution provides a robust, albeit empirical, description of the grain-size distribution in many different systems, the training set is representative of microstructures with a wide spectrum of grain sizes consistent with normal grain growth conditions. After training, U-Net was employed to segment the images in the test set considered here. Prior to analyzing these 512×512 test images, they were individually cropped to 480×480 pixels to remove unphysical border lines and then subjected to periodic boundary conditions. (The use of periodic boundary conditions here is not necessary but facilitates comparisons with idealized microstructures comprising, for example, periodically repeated square grains.) As the pixel data values lie on the interval $\left[0,1\right]$, it was convenient to binarize the data to highlight the grain-boundary pixels from the background. The reconstructed images in the test set were then analyzed as a function of the epoch number using the methodology described below.

### 2.3. Methodology for Nearest-Neighbor Analysis

The grain-boundary network is characterized stereologically in terms of the spatial statistics of the pixels comprising it. More specifically, the grain-boundary pixel locations are regarded as a spatial point process [20] that can be described through a hierarchy of *n*-point correlation functions [21], $\xi^{\left(n\right)}$, that reflect the positional associations among the pixels. While one can sometimes determine these correlation functions for small *n*, for larger values of *n*, the calculations become increasingly tedious, and it is advantageous to work instead with the moments of the *k*-th nearest-neighbor distributions.

To see that these neighbor distributions embody $\xi^{\left(n\right)}$ and therefore reflect important spatial correlations associated with the grain-boundary network, consider a point process in *d*-dimensions with a density $\rho_{d}$ and described by the *n*-point correlation functions, $\xi^{\left(n\right)}$, such that $\xi^{\left(0\right)}=0$ and $\xi^{\left(1\right)}=1$. These correlation functions reflect, for example, the tendency of points to cluster or, alternatively, to be effectively repelled from one another. To determine the probability of finding a certain number of neighbors in a volume $V_{d}$, one starts with the generating function [21,22]

(1) $$G\left(z\right)=\exp\left[\sum_{k=1}^{\infty }\frac{\rho_{d}^{k}{\left(z-1\right)}^{k}}{\Gamma (k+1)}\times \int_{V_{d}}\ldots \int_{V_{d}}d^{d}r_{1}\ldots d^{d}r_{k}\;\xi^{\left(k\right)}\left({\vec{r}}_{1},\ldots ,{\vec{r}}_{k}\right)\right],$$

where Γ is the gamma function [23], from which one can find the probability of finding *k* neighbors (k=1,2,…) in this volume via

(2) $$P_{k}=\frac{1}{\Gamma (k+1)}{\left[\frac{d^{k}}{dz^{k}}G\left(z\right)\right]}_{z=0}.$$

Thus, as expected, the probability of finding neighboring points within the same volume is determined by the correlations among these points and, consequently, the average neighbor distances, $\langle r_{k}\rangle$, and higher-order moments, $\langle r_{k}^{m}\rangle \left(m>1\right)$, embody these correlations as well. Thus, measures that compare reconstructed boundary networks that are based on these moments will reflect differences in pixel spatial correlations between distinct networks. This observation is the basis for the use of learning functions to quantify differences in network topology, as described below.

To illustrate the use of the neighbor distributions in this context, consider first a Poisson point process in *d* spatial dimensions with associated intensity $\rho_{d}$. The probability of finding k−1 points drawn from this distribution with an intensity $\rho_{d}$ in a hyperspherical region of radius *r* and the *k*-th point between *r* and r+dr is given by

(3) $$p_{k}\left(r\right)dr=\rho_{d}\frac{{\left[\rho_{d}V_{d}\left(r\right)\right]}^{k-1}}{\Gamma (k)}\;\exp\left(-\rho_{d}V_{d}\left(r\right)\right)\;S_{d-1}\left(r\right)\;dr,$$

where, in this case, the *d*-dimensional hyperspherical volume, $V_{d}\left(r\right)$, and (*d*-1)-dimensional surface area, $S_{d-1}\left(r\right)$, are given, respectively, by [24]

(4) $$\begin{array}{c} V_{d}\left(r\right)=\frac{\pi^{d/2}r^{d}}{\Gamma (\frac{d}{2}+1)},\\ S_{d-1}\left(r\right)=\frac{dV_{d}\left(r\right)}{dr}=\frac{2\pi^{d/2}r^{d-1}}{\Gamma (\frac{d}{2})}. \end{array}$$

The *m*-th moment of this distribution for k=1,2,… is given by

(5) $$\langle r_{k}^{m}\rangle =\int_{0}^{\infty }dr\;r^{m}p_{k}\left(r\right)={\left[\frac{1}{\rho_{d}}\right]}^{m/d}C\left(d,m\right)\frac{\Gamma (k+\frac{m}{d})}{\Gamma (k)},$$

where $C\left(d,m\right)={\left[\Gamma \left(\frac{d}{2}+1\right)/\pi^{d/2}\right]}^{m/d}$. Moreover, for the calculation of a neighbor-based cross-entropy, it is also useful to note that the average of the logarithm of the *m*-th moment can be obtained from the previous results via

(6) $$\frac{d\langle r_{k}^{m}\rangle }{dm}{|}_{m=0}=\langle \ln\left(r_{k}\right)\rangle =\frac{1}{d}\left[\psi \left(k\right)+\ln\left[\frac{\Gamma (\frac{d}{2}+1)}{\rho_{d}\pi^{d/2}}\right]\right],$$

where $\psi \left(k\right)$ is the digamma function [23].

For d=2, the first two moments of the distributions for k=1,2,… are given by

(7) $$\begin{array}{c} \langle r_{k}\rangle ={\left[\frac{1}{\rho_{2}\;\pi }\right]}^{1/2}\frac{\Gamma (k+\frac{1}{2})}{\Gamma (k)},\\ \langle r_{k}^{2}\rangle =\left[\frac{1}{\rho_{2}\;\pi }\right]k. \end{array}$$

These moments constitute a benchmark for comparison with the moments of the boundary pixel distributions, the latter calculated from U-Net processed micrographs.

### 2.4. Learning Functions

While some statistical tests exist to compare the moments of neighbor distributions [25,26], for the pixelized data used here, it is more convenient to construct other comparators to assess the rate at which the microstructural information embodied in the grain-boundary network is learned over the course of many U-Net epochs, *t*. For this purpose, two comparators, hereafter called learning functions, are constructed to emphasize their utility in this context. The first learning function, $L_{1}\left(t\right)$, is obtained from the sum over the neighbors of the moment differences between the current epoch number *t* and the final epoch number $t_{f}$. More specifically, after this sum is scaled by its maximum value, obtained using the starting epoch number, $t_{0}$, and the final epoch number, $t_{f}$, we define the ratio as

(8) $$L_{1}\left(t,t_{0},t_{f},m\right):=\frac{\sum_{k=1}^{k_{end}}\left[\langle r_{k}^{m}\rangle \left(t_{f}\right)-\langle r_{k}^{m}\rangle \left(t\right)\right]}{\sum_{k=1}^{k_{end}}\left[\langle r_{k}^{m}\rangle \left(t_{f}\right)-\langle r_{k}^{m}\rangle \left(t_{0}\right)\right]},$$

where $k_{end}$ is the final neighbor number considered and $0\leq L_{1}\leq 1$. It is of particular interest to compare the evolution of the U-Net cross-entropy loss function, l(t), with $L_{1}\left(t\right)$ to quantify the rate at which microstructural features are learned during U-Net boundary network formation, as summarized in Section 3 below.

The second learning function, $L_{2}\left(t\right)$, is related to a scaled cross-entropy based on the logarithms of the moments of the *k*-th neighbor distributions. As this comparator incorporates the structure of a grain-boundary network, we refer to it as a scaled microstructural cross-entropy. We note that the first moment of such distributions has been used in other fields to estimate, for example, the conformational and hydration entropies of molecules [27]. In this case, the construction of $L_{2}\left(t\right)$ is motivated by considering the cross-entropy between two probability density functions (pdfs) $p\left(x\right)$ and $q\left(x\right)$ associated with the values x of the random variable X, $H\left(p,q\right)$ [28]. The cross-entropy can be written in terms of the entropy associated with $p\left(x\right)$, $H\left(p\right)$, and the Kullback–Leibler (K-L) divergence, $D\left(p||q\right)$, [29] as

(9) $$H\left(p,q\right)=H\left(p\right)+D\left(p||q\right),$$

where the K–L divergence can be interpreted as the statistical distance between the two pdfs. In this context, we take *p* and *q* as the pdfs associated with the pixel locations in two (typically different) microstructures.

Several authors have shown that estimates of the K–L divergence can be calculated using $\langle \ln\left(r_{k}\right)\rangle$ [28,30]. To obtain the K–L divergence for microstructural comparison, one draws a set of *N* samples of pixel locations $\left\{X_{1},\ldots ,X_{N}\right\}$ from a particular microstructure generated at epoch number *t* and another set of *M* samples $\left\{Y_{1},\ldots ,Y_{N}\right\}$ from a fully reconstructed reference microstructure at time $t_{f}$. Then, if $\epsilon_{i}\left(k\right)$ is the Euclidean distance between $X_{i}$ and its *k*-th neighbor in $\left\{X_{1},\ldots ,X_{i-1},X_{i+1},\ldots ,X_{N}\right\}$ and $\nu_{i}\left(k\right)$ is the Euclidean distance between $X_{i}$ and its *k*-th neighbor in $\left\{Y_{1},\ldots ,Y_{N}\right\}$, then an estimator of the K–L divergence is given by

(10) $$\hat{D}\left(t,t_{f},k\right)=\frac{d}{\alpha }\sum_{i=1}^{\alpha }\ln\frac{\nu_{i}\left(k\right)}{\epsilon_{i}\left(k\right)}+\ln\left(\frac{\beta }{\alpha -1}\right),$$

where β (α) denotes max(M,N) (min(M,N)). Using this estimator, the second learning function is a scaled microstructural entropy (K–L divergence) defined as

(11) $$L_{2}\left(t,t_{0},t_{f},k\right):=\frac{\hat{D}\left(t,t_{f},k\right)}{\hat{D}\left(t_{0},t_{f},k\right)},$$

so that $0\leq L_{2}\leq 1$. Again, as above, it is of interest to compare the evolution of the U-Net binary cross-entropy loss function, l(t), with the microstructural cross-entropy, $L_{2}\left(t\right)$, as the former is based on a pixel-to-pixel comparison while the latter embodies a comparison of microstructural features and thereby quantifies the rate at which these features are learned.

## 3. Results

**Figure 2:**
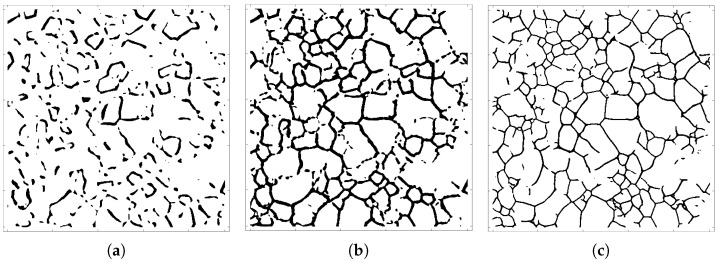
U-Net-traced microstructures for different epoch numbers: (**a**) 20, (**b**) 50, and (**c**) 295. Note the emergence of a connected grain-boundary network as the number of epochs increases.

As noted above, U-Net was employed to segment TEM micrographs obtained from a sputter-deposited Al thin film. Figure 2 shows the U-Net-traced microstructures for different epoch numbers. As is evident in the figure, while the grain-boundary network only begins to emerge after 20 epochs, a substantially more complete network skeleton is already present after 50 epochs, and this incomplete network subsequently evolves to fill in missing boundary segments as the number of epochs increases. To quantify the extent to which the topology of the boundary network is accurately reflected during reconstruction as a function of the epoch number, we use the two learning functions described above.

**Figure 3:**
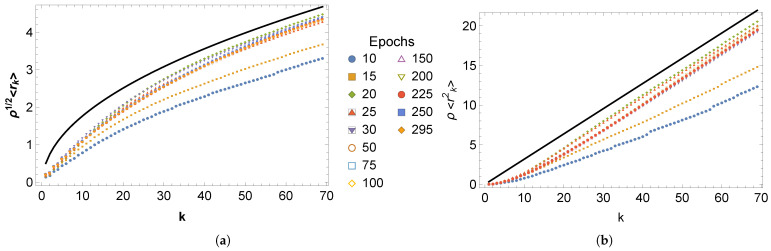
(**a**) The dimensionless first moment, $\rho_{2}^{1/2}\langle r_{k}\rangle \left(t\right)$, versus the neighbor number, *k*, for many epochs, *t*. The epoch numbers after which each curve was compiled are shown in the legend. The results for neighbors up to $k_{end}=70$ are presented. In addition, the results for a spatially random distribution of pixels are shown as a solid black line (see Equation (7)). (**b**) The dimensionless second moment, $\rho_{2}\langle r_{k}^{2}\rangle \left(t\right)$, versus the neighbor number, *k*, for many epochs, *t*. (see Equation (7)).

The learning function $L_{1}\left(t,t_{0},t_{f},m\right)$ is constructed from the information contained in the moments of the *k*-th nearest-neighbor distributions (see Equation (8)). Figure 3a,b show the dimensionless moments $\rho_{2}^{1/2}\langle r_{k}\rangle \left(t\right)$ and $\rho_{2}\langle r_{k}^{2}\rangle \left(t\right)$, respectively, as functions of the neighbor number, *k*, for many epochs, *t*, for microstructures generated using U-Net. Also shown for comparison are the results for a spatially random distribution of pixels (see Equation (7)). As is evident in the figures, as the epoch number increases, both $\rho_{2}^{1/2}\langle r_{k}\rangle \left(t\right)$ and $\rho_{2}\langle r_{k}^{2}\rangle \left(t\right)$ eventually converge to the corresponding limiting results (although not monotonically). In addition, the dimensionless moments differ considerably from those associated with a spatially random distribution for small *k*, since in this regime, neighboring pixels are arrayed approximately on line segments rather than distributed in 2d. Note that, in general, the dimensionless distance to the *k*-th neighbor is less than the corresponding distance for a random 2d distribution of pixels due to the more compact structure of the boundary network created by U-Net. In general, the curvature of $\rho_{2}^{1/2}\langle r_{k}\rangle$ as a function of *k* can be related to a transition from 1d to 2d behavior, and, as outlined in Section 4, one can extract an approximate average grain diameter from this plot.

**Figure 4:**
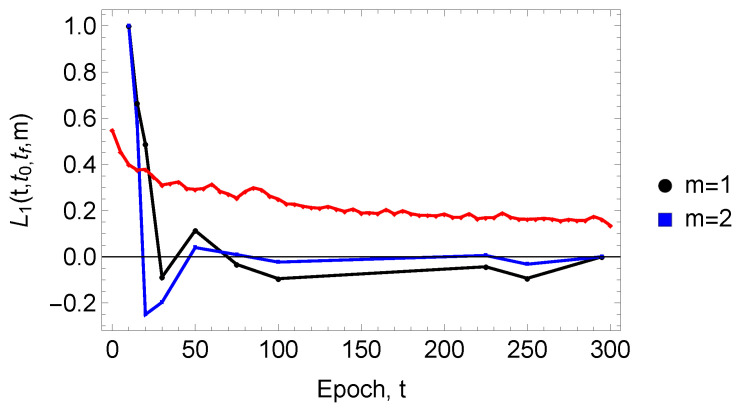
Comparison of the loss function l(t) (shown as a red line) with $L_{1}\left(t,t_{0},t_{f},m\right)$ (see Equation (8)) as a function of *t*, the latter for the first two moments, m=1,2 at a starting epoch number $t_{0}=15$ and a final epoch number $t_{f}=295$. The results are based on $k_{end}=70$ neighbors. It should be noted that one can also use a ground-truth image instead of the image corresponding to $t_{f}=295$. This ground truth can be obtained, for example, through hand-tracing of the microstructure [10].

From this information, we can compare and contrast the U-Net cross-entropy loss function, l(t), and $L_{1}\left(t,t_{0},t_{f},m\right)$ to determine the rate at which microstructural characteristics are learned during the U-Net boundary network construction. Figure 4 presents this comparison as a function of *t* for the first two moments, m=1,2. One can see in the figure that, as quantified by $L_{1}$, just after about 75 epochs, the U-Net-generated microstructural network reflects most of the important topological features of the fully reconstructed network. This behavior can be contrasted with that of l(t), which evinces a much slower decay with the epoch number. The decay rate for l(t) is approximately $3.3\times 10^{-3}$ epochs^−1^. While it is tempting to interpret negative values of $L_{1}$ as a negative correlation, particularly for m=2, such negative values may result from an accumulation of statistical uncertainties in the second moments of the neighbor distributions. Finally, if one regards l(t) and $L_{1}$ as pseudo-time-series data, the learning rate vis-à-vis l(t) can be obtained in terms of a cross-correlation function.

**Figure 5:**
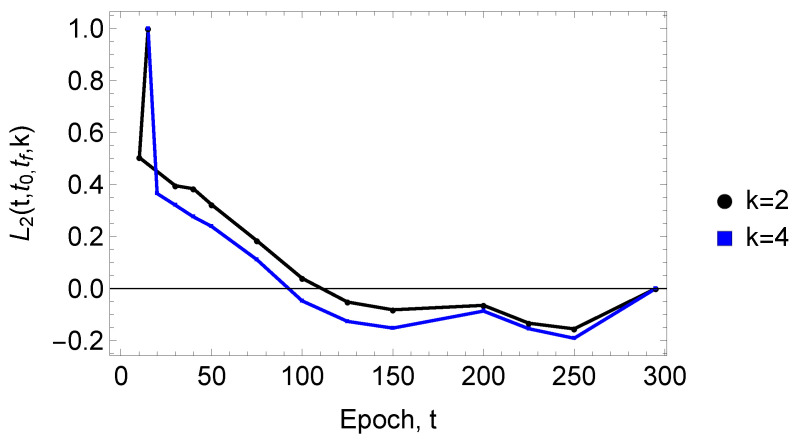
The dependence of the scaled microstructural cross-entropy, $L_{2}\left(t,t_{0},t_{f},k\right)$ (see Equation (11)) as a function of *t* for neighbors k=2,4 at a starting epoch number $t_{0}=10$ and a final epoch number $t_{0}=295$. The results are based on $k_{end}=70$ neighbors. The somewhat faster decrease in $L_{2}$ for k=4 may be specific to the microstructural network considered here rather than a general trend. In future work, it would be interesting to examine the behavior of $L_{2}$ as a function of *k* for various thin-film microstructures.

We next examine the evolution of the scaled microstructural cross-entropy, $L_{2}\left(t,t_{0},t_{f},k\right)$, as depicted in Figure 5, for nearest neighbors k=2 and k=4 (Given that $\nu_{i}\left(1\right)=0$ when the same pixel coordinates are in a given microstructure and the reference structure at epoch number $t_{f}$, we omit consideration of the first neighbor, k=1.). The spatial distribution of these nearest neighboring pixels is highly revealing. As is evident from the figure, after approximately 100 epochs, the principal characteristics of the microstructural network have been recovered by U-Net. As these nearest neighbors primarily describe pixel correlations on short boundary segments, these segments are, perhaps unsurprisingly, key to boundary network construction. These results provide additional evidence that microstructural network correlations develop early in the U-Net segmentation process.

## 4. Discussion and Conclusions

The aim of this work is to examine what is learned during U-Net-based segmentation of the microstructure of a material. We assessed the rate of microstructural learning associated with the segmentation of micrographs using two learning functions based on the moments of the *k*-th nearest-neighbor pixel distributions. These functions embody the spatial correlations among the pixels that comprise the grain-boundary networks that emerge during segmentation and are straightforward to calculate. It was found that the structure of these networks emerges after relatively few U-Net epochs, indicating that important microstructural features (e.g., grain size, network topology, etc.) are learned early during the segmentation process (as measured in epochs). In short, the microstructural learning rate is large relative to that associated with the U-Net binary, cross-entropy loss function.

To provide statistical validation for the claim that salient microstructural features emerge early in the U-Net reconstruction, we conducted paired *t*-tests for *k*-moment differences across epochs to assess the degree to which moment differences are significant. More specifically, we performed hypothesis tests for two pairs of dimensionless first moments: the first at epochs $t_{1}=10$ and $t_{2}=295$ and the second at epochs $t_{1}=75$ and $t_{2}=295$. For each pair of moments, we compared the null hypothesis $H_{0}:\sqrt{\rho_{2}}\langle r_{k}\rangle \left(t_{1}\right)-\sqrt{\rho_{2}}\langle r_{k}\rangle \left(t_{2}\right)=0$ with the alternative hypothesis $H_{A}:\sqrt{\rho_{2}}\langle r_{k}\rangle \left(t_{1}\right)-\sqrt{\rho_{2}}\langle r_{k}\rangle \left(t_{2}\right)\neq 0$. Under the assumption that the test statistic based on a pooled variance follows a t-distribution, we found that the corresponding p-values, p(k)≈0, for all *k*, an unsurprising result given the separation between the corresponding curves in Figure 3. In contrast, in the second case, it was found that p(k)>0.05 for all *k* (except for k=1). As one therefore cannot reject $H_{0}$ at the 5% significance level, this result suggests that the first moments are likely the same over nearly all values of *k* and that the microstructural networks at epochs 75 and 295 are essentially the same. The results from these tests should, however, be interpreted with some care, as samples from different epochs are not independent.

We note that some useful microstructural information can be inferred directly from the dependence of the pixel moment, $\langle r_{k}\rangle$, on the neighbor number *k*. As an illustration, consider a simplified 2*d* microstructure consisting of square grains, each with a side length ℓa on a square grid with pixels of side length *a*. For a fixed, large value of *k*, one expects that $\langle r_{k}\rangle \propto 1/\sqrt{\rho_{2}}$, where, in this case, $1/\sqrt{\rho_{2}}=\ell a/\sqrt{\left(2\ell -1\right)}\approx a\sqrt{\ell /2}$ for large *ℓ*. If one then uses Equation (7) as an approximation for $\langle r_{k}\rangle$ in general, one finds that, for large *k*,

(12) $$\langle r_{k}\rangle \approx {\left[\frac{k}{\pi }\right]}^{1/2}\frac{\ell a}{\sqrt{\left(2\ell -1\right)}}\left(1-\left(\frac{1}{8k}\right)\right),$$

where an asymptotic expansion is used for the gamma function. Equation (12) can be used to estimate the average grain size, ℓa. For example, for the last U-Net-constructed image analyzed above (i.e., epoch number 295), using this equation, one finds that ℓ≈44.7a, and so, using the pixel size a=1.6 nm associated with this image, ℓa≈71.6 nm. This result is relatively insensitive to the value of *k* for 50<k<70. (The effective circular-grain diameter for the corresponding ground-truth microstructure was found to be 100.6 nm. One could relax the circular-grain assumption by, for example, fitting individual grains with ellipses to account for different grain aspect ratios and then defining the grain diameter as the average of the lengths of the semi-major and semi-minor axes. Similarly, one can extend our square-grain model by using different-sized squares or rectangles to represent grains.)

As indicated above, the moments given in Equations (5)–(7) are used simply for comparison with those calculated directly from the data. In other words, the calculation of the moments from the pixelated data does not involve the continuous spatial assumption used to derive the moments in Equations (5)–(7). Nevertheless, it is useful to provide approximate correction factors that connect the pixelated calculations with their continuous counterparts [31]. For example, for d=2, the number of neighbors, *k*, within a circle with radius $\langle r_{k}\rangle$ is given by $k\approx \rho_{2}\pi {\langle r_{k}\rangle }^{2}$. Taking into account the pixels that lie along the circumference of this circle, one can set bounds on the radius. One finds that

(13) $$\sqrt{\frac{k}{\rho_{2}\pi }}\;\left[1-\sqrt{\frac{2\pi \rho_{2}a^{2}}{k}}\right]<\langle r_{k}\rangle <\sqrt{\frac{k}{\rho_{2}\pi }}\;\left[1+\sqrt{\frac{2\pi \rho_{2}a^{2}}{k}}\right].$$

Thus, for large *k*, $\langle r_{k}\rangle \approx \sqrt{k/\rho_{2}\pi }$, with the corrections given by Equation (13).

The approach outlined here for boundary network reconstruction can be improved by combining the image information obtained from different sample tilts of the same field of view. The underlying issue is that the contrast between neighboring grains changes as the specimen is tilted such that, at any given tilt, a number of boundaries will likely be out of contrast. To mitigate this information loss, if multiple images corresponding to different tilts are available, one can form a blended image using a logical OR operation that yields a boundary pixel location if a boundary pixel is found at any tilt [9]. The blended image will, therefore, contain boundary information that may be absent in an image at a particular tilt. The evolution of this blended image can then be tracked as a function of the epoch number.

Finally, given the utility of the functions $L_{1}$ and $L_{2}$ in capturing microstructural learning, it is reasonable to ask whether the existing binary, cross-entropy loss function can be augmented with $L_{1}$ and/or $L_{2}$. Given the rapid convergence of the learning functions with time, it would be best not to employ them solely as a loss function but rather to construct a multi-objective loss function comprising $\ell \left(t\right)$ and either $L_{1}$ or $L_{2}$. The incorporation of these learning functions can profitably enhance the convergence of U-Net segmentation. The creation of such a compound loss function is the subject of the current study.

## Data Availability

The authors will make available, upon request, the data used in this work. It is understood that the data provided will not be for commercial use.

## References

[1] Hansen N. (2004). Hall-Petch relation and boundary strengthening. Scr. Mater..

[2] Hanaor D.A.H., Xu W., Ferry M., Sorrell C.C. (2012). Abnormal grain growth of rutile TiO_2_ induced by ZrSiO_4_. J. Cryst. Growth.

[3] Zhu M.-L., Xuan F.-Z. (2010). Correlation between microstructure, hardness and strength in HAZ of dissimilar welds of rotor steels. Mat. Sci. Eng. A.

[4] Yoo E., Moon J.H., Sang Y., Kim Y., Ahn J.-P., Kim Y.K. (2020). Electrical resistivity and microstructural evolution of electrodeposited Co and Co-W nanowires. Mater. Charact..

[5] Barmak K., Lu X., Darbal A., Nuhfer N.T., Choi D., Sun T., Warren A.P., Coffey K.R., Toney M.F. (2016). On twin density and resistivity of nanometric Cu thin films. J. Appl. Phys..

[6] Bedu-Amissah K., Rickman J.M., Chan H.M., Harmer M.P. (2007). Grain-boundary diffusion of Cr in pure and Y-doped alumina. J. Am. Ceram. Soc..

[7] Wang C.-M., Cho J., Chan H.M., Harmer M.P., Rickman J.M. (2001). Influence of dopant concentration on creep properties of Nd_2_O_3_-doped alumina. J. Am. Ceram. Soc..

[8] Cho J., Wang C.M., Chan H.M., Rickman J.M., Harmer M.P. (2001). Improved tensile creep properties of yttrium- and lanthanum-doped alumina: A solid solution effect. J. Mater. Res..

[9] Carpenter D.T., Rickman J.M., Barmak K. (1998). A methodology for automated quantitative microstructural analysis of transmission electron micrographs. J. Appl. Phys..

[10] Patrick M.J., Eckstein J.K., Lopez J.R., Toderas S., Levine S., Rickman J.M., Barmak K. (2023). Automated grain boundary detection for bright-field transmission electron microscopy images via U-Net. Microsc. Microanal..

[11] Patrick M.J., Eckstein J.K., Lopez J.R., Toderas S., Levine S., Barmak K. (2023). U-Net implementation for high throughput grain boundary detection in bright field TEM micrographs: Toward in situ grain growth studies. Microsc. Microanal..

[12] Williams D.B., Carter C.B. (2010). Transmission Electron Microscopy: A Textbook for Materials Science.

[13] Jocher G., Chaurasia A., Qiu J. (2023). Ultralytics YOLOv8.

[14] Jadon S. A survey of loss functions for semantic segmentation. Proceedings of the 2020 IEEE Conference on Computational Intelligence in Bioinformatics and Computational Biology (CIBCB).

[15] Ciampiconi L., Elwood A., Leonardi M., Mohamed A., Rozza A. (2023). A survey and taxonomy of loss functions in machine learning. arXiv.

[16] Barmak K., Rickman J.M., Patrick M.J. (2024). Advances in experimental studies of grain growth in thin films. JOM.

[17] Siddique N., Paheding S., Elkin C.P., Devabhaktuni V. (2021). U-net and its variants for medical image segmentation: A review of theory and applications. IEEE Access.

[18] Ronneberger O., Fischer P., Brox T. (2015). U-Net: Convolutional networks for biomedical image segmentation. Medical Image Computing and Computer-Assisted Intervention (MICCAI).

[19] Gao B., Pavel L. (2017). On the properties of the softmax function with application in game theory and reinforcement learning. arXiv.

[20] Diggle P.J. (2014). Statistical Analysis of Spatial and Spatio-Temporal Point Patterns.

[21] van Kampen N.G. (2007). Stochastic Processes in Physics and Chemistry.

[22] Banerjee A., Abel T. (2021). Nearest neighbour distributions: New statistical measures for cosmological clustering. Mon. Not. R. Astron. Soc..

[23] Arfken G.B., Weber H.J. (2005). Mathematical Methods for Physicists.

[24] Luscombe J.H. (2018). Thermodynamics.

[25] Schilling M.F. (1986). Multivariate two-sample tests based on nearest neighbors. J. Am. Stat. Assoc..

[26] Friedman J.H., Steppel S. (1974). A Nonparametric Procedure for Comparing Multivariate Point Sets.

[27] Fogolari F., Borelli R., Dovier A., Esposito G. (2024). The kth nearest neighbor method for estimation of entropy changes from molecular ensembles. Wiley Interdiscip. Rev. Comput. Mol. Sci..

[28] Li S., Mnatsakanov R.M., Andrew M.E. (2011). k-nearest neighbor based consistent entropy estimation for hyperspherical distributions. Entropy.

[29] Cover T.M., Thomas J.A. (2006). Elements of Information Theory.

[30] Zhao P., Lai L. Analysis of k nearest neighbor KL divergence estimation for continuous distributions. Proceedings of the 2020 IEEE International Symposium on Information Theory (ISIT).

[31] Miyagawa M. (2012). An approximation for the kth nearest distance and its application to locational analysis. J. Oper. Res. Soc. Jpn..

